# Modeling of physical-mechanical and microbiological properties of tablets made of complex fluidized bed granules containing living yeast cells using common mixing rules

**DOI:** 10.1016/j.ijpx.2025.100423

**Published:** 2025-10-23

**Authors:** Karl Vorländer, Lukas Bahlmann, Arno Kwade, Jan Henrik Finke, Ingo Kampen

**Affiliations:** a Technische Universität Braunschweig, Institute for Particle Technology, Volkmaroder Straße 5, 38104 Braunschweig, Germany; b Technische Universität Braunschweig, Center of Pharmaceutical Engineering, Franz-Liszt-Straße 35A, 38106 Braunschweig, Germany

**Keywords:** Probiotics, *Saccharomyces cerevisiae*, Tableting, Compressibility, Compactibility, Tabletability, Volumetric mixing rules

## Abstract

In order to administer probiotic microorganisms effectively, suitable dosage forms and production methods are required. These must be geared towards maintaining viability, which is essential for the health-promoting properties. In earlier studies, fluidized bed spray granulation with subsequent further processing into tablets showed promising results. The physical-mechanical and microbiological tablet properties were found to depend on the excipient. The occurrence of advantageous synergies was investigated by combining different excipients during granulation. Since mixed properties were largely observed, volume-weighted mixing rules were applied to predict the compressibility, compactibility and tabletability of single, binary and ternary carrier granules based on the tableting of the non-granulated excipients. For one of the three carriers investigated, the common model had to be extended by a correction term, whereas for the other two carriers, a very good prediction could be made directly. Similarly, the survival of the microorganisms in single-carrier granules was modeled and used to predict survival in binary and ternary mixed granules. In contrast, the prediction of the microbiological survival was less accurate. Overall, the combination of lactose and microcrystalline cellulose turned out to be overall advantageous for survival. However, this is due to the especially high survival during granulation and not during tableting. The previously identified dependence of survival on porosity reduction was confirmed for the more complex formulations and could be the basis for further development of models to predict survival during compaction.

## Graphical abstract

**Figure:**
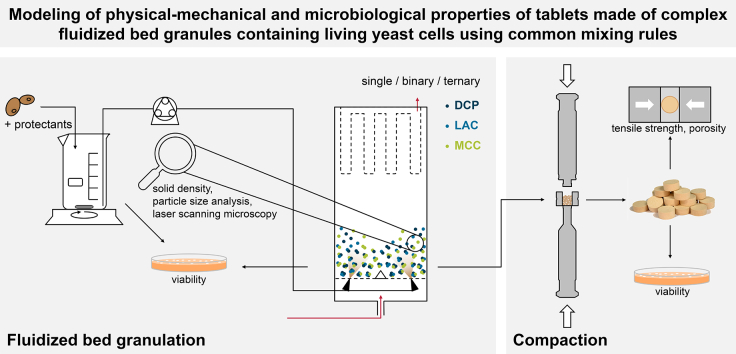

## Nomenclature

a: fit parameter
b: fit parameter
d: tablet diameter, mm
ER: elastic recovery
F: tablet breaking force, N
h: tablet height, mm
$h_{\min}$: minimal tablet height during compaction, mm
$k_{\text{apparent}}$: apparent Heckel constant, ${\mathrm{MPa}}^{-1}$
$k_{b}$: bonding capacity
$k_{\mathrm{SR},\varepsilon }$: tablet porosity-specific inactivation rate
$k_{\mathrm{SR},\sigma_{t}}$: tablet tensile strength-specific inactivation rate
$m_{t}$: tablet mass, g
n: number of components, number of replicates
$P_{Y}$: mean yield pressure, MPa
$R^{2}$: coefficient of determination
SR: survival rate, %
$SR_{\sigma_{t}=0}$: theoretical survival rate at zero tensile strength, %
$V_{i}$: solid volume of component i, $m^{3}$
$V_{\text{total}}$: total solid volume of formulation, $m^{3}$
x: particle size, μm
$x_{i}$: mass fraction
ε: tablet porosity
$\varepsilon_{\text{blend}}$: tablet porosity of blend calculated via mixing rule
$\varepsilon_{\text{experiment}}$: experimental tablet porosity
$\varepsilon_{\mathrm{fit}}$: fitted tablet porosity
$\varepsilon_{\text{in}-\mathrm{die}}$: minimal in-die porosity
$\varepsilon_{\min}$: minimal tablet porosity
$\varepsilon_{\text{model}}$: predicted tablet porosity
$\varepsilon_{\text{reducible}}$: reducible tablet porosity
$\rho_{s}$: solid density, $g\;{\mathrm{cm}}^{-3}$
$\sigma_{c}$: compression stress, MPa
$\sigma_{t}$: tablet tensile strength, MPa
$\sigma_{0}$: theoretical maximum tablet tensile strength at zero porosity, MPa
$\varphi_{i}$: bulk volume fraction
$\varphi_{a,i}$: surface area fraction
$\varphi_{s,i}$: solid volume fraction

## 1 Introduction

### 1.1 Formulation of tablets containing living microorganisms

Probiotic microorganisms can promote patient health when taken in sufficient quantities (Joint FAO/WHO Working Group, 2002). This requires suitable dosage forms and manufacturing processes that allow for gentle processing (Broeckx et al., 2016). While liquid dosage forms are easy to produce without critical stress on the microorganisms, their storage stability is usually limited. Although cooling can improve their stability, it is complex and expensive (Santivarangkna, 2016). Drying is a more convenient and cost-effective method for conservation compared to freezing (Lievense and van't Riet, 1993), which typically allows for even greater storage stability than cooling (Meryman, 1974). However, drying can result in a reduction of viability but the surviving cells can be converted into a dry, storable form and subsequently reactivated (Broeckx et al., 2016). Suitable processes and process parameters, as well as formulations that minimize stress to the microorganisms, are the basic prerequisites to ensure that the inactivation is reversible. Various processes are suitable for life-preserving drying, such as classic freeze-drying, spray drying, or fluidized bed spray granulation (Broeckx et al., 2016; Lievense and van't Riet, 1993).

Although dried powders or granules can be administered directly or packaged in capsules, tableting is usually preferred. Loose granules expose microorganisms to harsh stomach conditions more than other dosage forms. Packaging into capsules is also more expensive than tableting. However, tableting is associated with pressure and shear stresses that can be lethal to microorganisms, leading to irreversible inactivation. This effect increases with higher compression stress (Ayorinde et al., 2011; Blair et al., 1991; Chan and Zhang, 2002; Silva et al., 2013; Fassihi and Parker, 1987; Muller et al., 2014; Nagashima et al., 2013; Plumpton et al., 1986a, Plumpton et al., 1986b; Poulin et al., 2011; Stadler and Viernstein, 2001; Vorländer et al., 2025a, Vorländer et al., 2025b; Vorländer et al., 2024; Vorländer et al., 2023a; Vorländer et al., 2023c; Vorländer et al., 2023d; Vorländer et al., 2023b; Vorländer et al., 2020). Therefore, process and formulation parameters must be carefully selected. Especially the formulation was addressed in many studies dealing with compaction of viable microorganisms dried by lyophilization, spray-drying or granulation (Ayorinde et al., 2011; Azhar and Munaim, 2021; Blair et al., 1991; Brachkova et al., 2009; Byl et al., 2018; Chan and Zhang, 2002; Silva et al., 2013; Fassihi and Parker, 1987; Hoffmann and Daniels, 2019; Huq et al., 2016; Klayraung et al., 2009; Maggi et al., 2000; Muller et al., 2014; Nagashima et al., 2013; Oktavia et al., 2020; Plumpton et al., 1986a, Plumpton et al., 1986b; Poulin et al., 2011; Sánchez et al., 2018; Shu et al., 2020; Stadler and Viernstein, 2003; Stadler and Viernstein, 2001; Villena et al., 2015; Vorländer et al., 2023a; Vorländer et al., 2023c; Vorländer et al., 2023d; Vorländer et al., 2023b; Vorländer et al., 2020).

Although fluidized bed granulation is a promising method for drying microorganisms (Vorländer et al., 2023a), it has been little used and knowledge on the influence of the formulation on survival during tableting of granules is limited (Ayorinde et al., 2011; Blair et al., 1991; Fassihi and Parker, 1987; Hoffmann et al., 2020; Hoffmann and Daniels, 2019; Plumpton et al., 1986a, Plumpton et al., 1986b; Vorländer et al., 2025a, Vorländer et al., 2025b; Vorländer et al., 2023a; Vorländer et al., 2023c; Vorländer et al., 2023b) (in some of these publications wet granules were oven-dried). In previous studies by the same authors, the survival of microorganisms during tableting of granules prepared by fluidized bed spray granulation was found to depend on the carrier material used (Vorländer et al., 2025b; Vorländer et al., 2023a; Vorländer et al., 2023c; Vorländer et al., 2023b). This dependence was attributed to specific deformation characteristics of the carrier materials, and survival could be correlated with reduction of porosity during compression across formulations (Vorländer et al., 2023c; Vorländer et al., 2023b). However, granulation of carrier materials with microorganisms and protectants was also found to negatively affect properties such as compressibility, compactibility, and tabletability (Vorländer et al., 2025a, Vorländer et al., 2025b; Vorländer et al., 2023b). To what extent the combination of different deforming materials during granulation has positively or negatively reinforcing effects on physical-mechanical and microbiological tablet properties has not yet been investigated. This is addressed in the present study. For this purpose, granules based on binary mixtures and a ternary mixture of three dry binders (dicalcium phosphate (DCP), lactose (LAC) and microcrystalline cellulose (MCC)) are tableted and the physical-mechanical tablet properties as well as the viability of the microorganisms are analyzed.

### 1.2 Modeling physical-mechanical properties of multi-component tablets

Predicting the properties of tablets made from blends of different components based on the characterization of the tableting properties of the pure components is the subject of numerous studies (Puckhaber et al., 2023). The development of reliable models is crucial for efficient formulation development with minimal experimental effort. To this end, for the prediction of powder blend tableting behavior it is necessary to be able to describe both the compressibility and the compactibility of the pure components precisely using suitable models. These are then combined in a suitable way to predict the compressibility, compactibility and tableting behavior of multi-component tablets.

For the compressibility of binary mixtures of microcrystalline cellulose (MCC) and lactose (LAC), a linear dependence of tablet porosity on the mass fraction of the components has been reported (Busignies et al., 2006). Furthermore, it was postulated that the compression of the components occurs independently of each other (Frenning et al., 2009). Based on this, the compressibility of binary pellet mixtures was fitted using Kawakita's compressibility model (Kawakita and Lüdde, 1971), and a linear correlation was found between the effective Kawakita parameters obtained and the volume fraction of the components (Frenning et al., 2009). However, these parameters depend on the initial bulk volume and are not intrinsic material properties (Mazel et al., 2011). Therefore, Mazel et al. took a different approach and used the Kawakita parameters of the individual components to calculate the respective porosities of these and successfully predicted the porosity of the binary mixture during compression by volumetric weighting (Mazel et al., 2011). This approach allowed the prediction of the compressibility of physical powder mixtures of up to five components (Busignies et al., 2012).

One approach to predict the tensile strength of binary mixtures of one well and one poorly compactable component is based on the percolation theory (Kuentz and Leuenberger, 2000). However, this model is only valid for a small compression stress range up to 60 MPa (Kuentz and Leuenberger, 2000). In further studies, the model was extended for the combination of two components with good compactibility (Ramírez et al., 2004). However, the application requires experimental data of the binary mixture and is not suitable for prediction on the basis of single component investigations (Wu et al., 2005). Therefore, Wu et al. used the Ryshkewitch-Duckworth (Duckworth, 1953; Ryshkewitch, 1953) model to describe the compactibility of the individual components and were able to predict the compactibility of binary (Wu et al., 2005) and multi-component tablets (Wu et al., 2006) by taking the volume fraction into account. Reynolds et al. enabled improved prediction accuracy by using a geometric mean rule (Reynolds et al., 2017).

Based on this, the present study investigates the possibility of predicting the physicomechanical properties of granules with living microorganisms using the compaction of the carrier materials and spray-dried microorganisms. In order to also predict the survival of the microorganisms, the application of analogous volume-weighted mixing rules for the microbiological tablet properties is examined.

## 2 Materials and methods

### 2.1 Materials

In the present study, the yeast *Saccharomyces cerevisiae* (Lallemand-DHW GmbH, Vienna, Austria) was used as a model microorganism. Dicalcium phosphate (DCP, DI-CAFOS A150, Chemische Fabrik Budenheim KG, Budenheim, Germany), lactose (LAC, Granulac 70, MEGGLE GmbH & Co. KG, Wasserburg am Inn, Germany) and microcrystalline cellulose (MCC, Vivapur 102, J. Rettenmaier & Söhne GmbH + Ko KG) served as carrier materials. Trehalose dihydrate (FormMed HealthCare AG, Frankfurt am Main, Germany) and skim milk powder (Carl Roth GmbH + Co. KG, Karlsruhe, Germany) were used as protective additives. Magnesium stearate (MgSt, MAGNESIA GmbH, Lüneburg, Germany) was added as a lubricant during tableting. To determine viability, a phosphate-buffered saline solution (1.6 g L^−1^ NaCl, 0.04 g L^−1^ KCl, 0.284 g L^−1^ Na_2_HPO_4_, 0.054 g L^−1^ KH_2_PO_4_, pH 7.4; Sigma-Aldrich Chemie GmbH, München, Germany) was prepared. The agar plates used for this purpose consisted of 10 g L^−1^ of yeast extract, 20 g L^−1^ of peptone ex casein, 22 g L^−1^ of glucose monohydrate and 15 g L^−1^ of Agar-Agar Kobe 1 (all from Carl Roth GmbH + Co. KG, Karlsruhe, Germany).

### 2.2 Fluidized bed spray granulation of *Saccharomyces cerevisiae*

In a fluidized bed granulator, a suspension of the yeast cells was sprayed onto different carrier particles. In a previous study of the same authors, these were either DCP, LAC and MCC. In the present study, three binary mixtures and one ternary mixture of these materials were used as carrier and starting material for fluidized bed granulation. The different materials were combined in equal weight proportions (1:1 and 1:1:1) and 1 kg of the mixture was used for granulation (Solidlab 2, Syntegon Technology GmbH, Waiblingen, Germany). Beside the yeast cells (18.8 wt.-% (cell dry weight, CDW)) and water, the granulation liquid consisted of the approved protective additives trehalose dihydrate (9.4 wt.-%) and skim milk powder (9.4 wt.-%) (Vorländer et al., 2023a, Vorländer et al., 2023b). After incubation at room temperature for 1 h, the suspension was sprayed onto the fluidized carrier particles. The inlet temperature was 50 °C and the air volume flow was set as required for sufficient fluidization. Due to the varying energy input, the pump rate of the suspension was set in such a way (up to 40 g/min) that excessively high bed temperatures (above 40 °C) were avoided. More details can be found elsewhere (Vorländer et al., 2023a, Vorländer et al., 2023b).

### 2.3 Spray drying of granulation liquid

In addition, the granulation liquid was dried by spray drying in a co-current flow (ProCepT 4 M8-TriX, PROCEPT nv, Zele, Belgium). In order to limit the thermal stress on the product, the product was removed after every 60 min of drying time. The inlet temperature was 100 °C, the volume flow of the drying air 0.3 m^3^/min, the mass flow of the cell suspension 2 g/min, the nozzle diameter 1.2 mm and the nozzle pressure 1.5 bar. In addition, 0.12 m^3^/min air was supplied to the cyclone to reduce the separation limit. Before the cell suspension was sprayed, water was sprayed for at least 15 min to bring the entire system to a state of equilibrium (temperature and humidity). The flow rate was reduced to 1.25 g/min when spraying the water because the proportion of water in the cell suspension was approx. 0.62. By this, the same mass flow of vaporizable water was sprayed as during the spraying of the cell suspension minimizing temperature and moisture changes in the spray dryer when switching from spraying water to spraying the cell suspension. These settings were identified as suitable in a previous study (Vorländer et al., 2023d).

### 2.4 Granule characterization

#### 2.4.1 Scanning electron microscopy

Samples of the granules were sputter-coated with platinum (LEICA EM ACE600, Leica Microsystems GmbH, Wetzlar, Germany) before scanning electron microscopy (SEM) images were taken (Helios G4 CX, FEI, Hilsboro, OR, United States; now Thermo Fisher Scientific).

#### 2.4.2 Particle size analysis

The particle size distributions of ungranulated carrier material and of the granules were analyzed by dynamic image analysis (QICPIC with GRADIS dispersing unit and VIBRI dosing unit, Clausthal-Zellerfeld, Germany). The analysis was performed in triplicate with a minimum of 100,000 particles analyzed each time.

#### 2.4.3 Solid density

The true density of the granules $\rho_{s}$ was determined by helium gas pycnometry (Ultrapyc 1200e, Quantachrome Instruments, Boynton Beach, FL, United States). The samples were weighed once and measured 10 times.

### 2.5 Tableting

Prior to tableting, the carrier LAC, the single carrier granules based on DCP and LAC and the binary mixed granules based on these two components, were lubricated with 0.5 wt.-% of MgSt. For this purpose, granules and lubricant were mixed for 2 min at 49 min^−1^ in a 3D shaker mixer (TURBULA, Willi A. Bachofen AG, Muttenz, Switzerland). DCP was lubricated using 1.0 wt.-% of MgSt. The tablets were produced using a compaction simulator (Styl'One evolution, Medelpharm, Beynost, France). A generic, symmetric compression profile with 90 ms given pressure increase/decrease time and 20 ms given dwell time was used. The compaction simulator was equipped with flat EUR-D tools with a diameter of 11.28 mm. The die was filled with about 450 mg of the granules using a paddle feeder. Compression stresses in the range of 50 to 300 MPa were applied displacement-controlled for the binary and ternary mix granules. The carrier particles and granules based solely either on DCP, LAC or MCC, were tableted during previous studies (Vorländer et al., 2023c; Vorländer et al., 2023b) where compression stresses up to 400 MPa were used. The tablets were stored in polyethylene bags at 2 °C for 24 h prior to characterization.

### 2.6 Tablet characterization

#### 2.6.1 Physical tablet properties

10 tablets were weighed and the diameter d and the height h were measured using a manual tablet tester (MultiTest 50, Sotax AG, Aesch, Switzerland). With this device, the breaking force F was determined under diametrical load (Ph. Eur. 9.3, 2.9.8). Breaking force, tablet diameter and height were used to calculate tablet tensile strength $\sigma_{t}$ (Carneiro and Barcellos, 1949, Fell and Newton, 1970):

(1) $$\sigma_{t}=\frac{2\;F}{\pi \;d\;h}$$

Together with the tablet weight $m_{t}$ and the solid density $\rho_{s}$ determined by helium gas pycnometry before compaction, height and diameter were used to calculate the porosity ε of the tablets as

(2) $$\varepsilon =1-\frac{m_{t}}{\rho_{s}\;\frac{\pi }{4}\;d^{2}\;h}$$

In addition, the apparent minimal in-die porosity $\varepsilon_{\text{in}-\mathrm{die}}$ was calculated with the same equation but with minimal tablet height $h_{\min}$ during compaction and the diameter of the die $d_{\mathrm{die}}$.

The elastic recovery was calculated as the axial change of tablet height as (Armstrong and Haines-Nutt, 1972)

(3) $$\mathrm{ER}=\frac{h-h_{\min}}{h_{\min}}$$

#### 2.6.2 Microbiological tablet properties

As described elsewhere, the number of viable microorganisms in the tablets and also the granules as a reference were determined as colony forming units (CFU) (Vorländer et al., 2023a). In brief, the tablets or 450 mg of the granules were suspended in 10 mL of the phosphate-buffered saline solution. After gentle stirring for 1 h at room temperature, serial dilutions were prepared and suitable concentrations were spread on agar plates. After incubation at 30 °C for approx. 30 h, the number of colonies formed was counted and the viability was calculated as (Vorländer et al., 2023a)

(4) $$\text{viability}\;\left[\mathrm{CFU}\;g_{\mathrm{CDW}}^{-1}\right]=\frac{\text{count of colonies}\;\left[\mathrm{CFU}\right]}{\text{plated concentration}\;\left[g_{\mathrm{CDW}}\;L^{-1}\right]\cdot \text{plated volume}\;\left[L\right]}$$

To calculate survival rates, initial and resulted viability were related to each other, for example for the tableting step:

(5) $$\text{survival rate}\;\left[\%\right]=\frac{\text{viability after compression}\;\left[\mathrm{CFU}\;g_{\mathrm{CDW}}^{-1}\right]}{\text{viability before compression}\;\left[\mathrm{CFU}\;g_{\mathrm{CDW}}^{-1}\right]}$$

## 3 Results and discussion

### 3.1 Granules

**Fig. 1:**
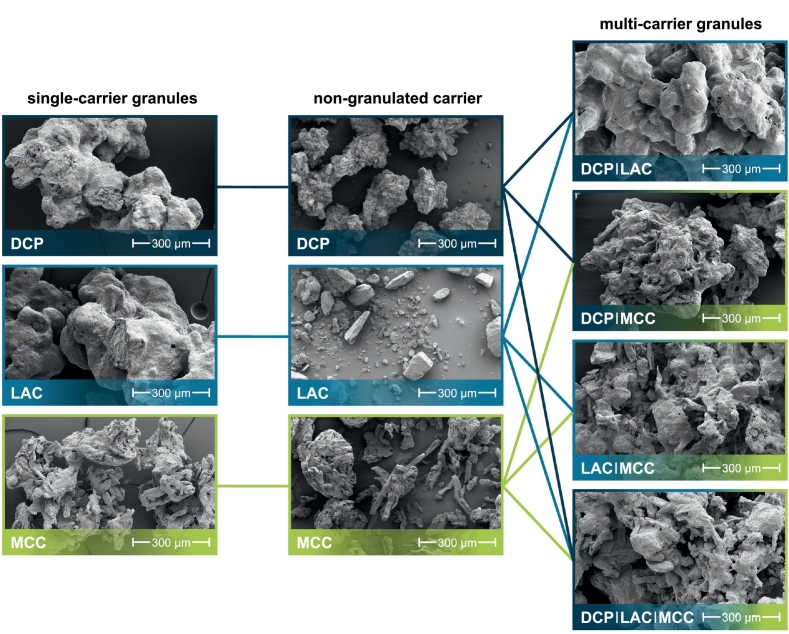
SEM images of the yeast granules based on single carrier materials (left), of non-granulated carrier materials (middle) and of yeast granules based on binary or ternary mixtures of the carrier materials (right). Yeast cells can be seen on the surface of the carrier particles (small oval elevations, partially embedded in a closed matrix of the protective additives). For a higher resolution, please refer to the digital version of the publication.

Seven different granules were produced (Fig. 1) for further processing into tablets. DCP, LAC and MCC as single excipients or the corresponding binary as well as the corresponding ternary mixture of these components served as carrier materials in the following granulations. The mixtures contained the respective components in equal proportions by mass. The mass fraction of yeast cells was $\left(0.144\pm 0.009\right)\;g_{\mathrm{CDW}}\;g_{\text{granules}}^{-1}$. An equal weight proportion of the granules consisted of the protective substances trehalose and milk powder. Due to the different solid densities of the carrier materials, the volume fractions differed (Table 1).

**Table 1:** Volumetric fractions of the various components of the different granules. Line sums not equal to one are the consequence of rounded values.

granules	volumetric fraction			
	yeast + additives	DCP	LAC	MCC
DCP	0.442	0.558	–	–
LAC	0.288	–	0.712	–
MCC	0.294	–	–	0.706
DCP/LAC	0.394	0.211	0.394	–
DCP/MCC	0.373	0.222	–	0.405
LAC/MCC	0.298	–	0.354	0.347
DCP/LAC/MCC	0.374	0.133	0.249	0.244

During granulation, the yeast cells and in particular the protective additives trehalose and milk powder act as binding agents, lead to an increase in particle size. The non-granulated starting materials show similar particle size distributions, with the average particle size of LAC being the lowest and MCC the highest (particle size distributions can be found in Fig. S1). Non-granulated DCP is characterized by the narrowest particle size distribution of the carriers, non-granulated LAC by the widest span (Table 2). LAC and DCP form granules that have almost identical particle size distributions with an average particle size of around 820 μm. The average particle size of the granules from the binary mixture DCP/LAC is slightly smaller at approx. 685 μm. The MCC granules have a significantly smaller average particle size of around 470 μm. Due to the high water absorption capacity of MCC, the formation of liquid bridges is limited here compared to DCP and LAC, which also reduces the formation of solid bridges (reduced agglomeration / granule growth). Due to the loose, fibrous structure of MCC, it is also conceivable that the granules formed are easier to separate again (increased attrition / breakage) (Fig. 1). In addition, the particle surface accessible to the granulation liquid is larger, which is why the layers on the particles are not quite as dense and thick (Fig. 1). The combinations of DCP/MCC, LAC/MCC or DCPLAC/MCC results in intermediate particle size distributions with an average particle size of around 620–670 μm (Fig. S1, Table 2). The average particle size increases slightly as the volume proportion of MCC in the granules decreases as the surface moisture increases. The survival rate during granulation was observed to be 19.7 ± 0.9 % in case of DCP granules, 13 ± 2 % in case of LAC granules and 10.3 ± 0.2 % in case of MCC granules. The survival rate for the granules based on the binary mixture DCP/MCC was 14.8 ± 0.7 %, while the survival rate during granulation of the other three mixtures was 6.6 ± 0.7 %. The different survival rates can essentially be attributed to different thermal stresses (duration and intensity) and drying kinetics (Vorländer et al., 2023a, Vorländer et al., 2023b).

**Table 2:** Characteristic particle sizes and span of particle size distributions.

material	$x_{10}\;\left[\mathrm{\mu m}\right]$	$x_{50}\;\left[\mathrm{\mu m}\right]$	$x_{90}\;\left[\mathrm{\mu m}\right]$	span = $\frac{x_{90}-x_{10}}{x_{50}}$
non-granulated				
DCP	110	185	281	0.92
LAC	75	156	283	1.33
MCC	96	214	369	1.27
granules				
DCP	498	817	1204	0.86
LAC	456	820	1234	0.95
MCC	281	469	685	0.86
DCP/LAC	365	685	1029	0.97
DCP/MCC	345	621	933	0.95
LAC/MCC	365	670	1023	0.98
DCP/LAC/MCC	411	667	987	0.86

### 3.2 Modeling of compressibility

Various models for describing the compressibility of powders are established in the literature (Gurnham and Masson, 1946; Heckel, 1961; Kawakita and Lüdde, 1971; Kawakita and Tsutsumi, 1966; Kuentz and Leuenberger, 1999; Reynolds et al., 2017; Vreeman and Sun, 2022; Zhao et al., 2006). In order to reliably predict the properties of tablets made from several components, models for the individual components are needed that show a very good correlation with the experimental data in the entire compression stress range to be evaluated. Various compressibility models were applied, with a model recently published by the same authors proving to be particularly suitable (Vorländer et al., 2025b). This is based on the long-established model by Heckel (Heckel, 1961), but is applied in exponential form and has an additional term that describes the minimum tablet porosity after ejection and elastic recovery of the tablets. This makes the model particularly suitable for modeling / fitting out-die tablet porosities.

(6) $$\varepsilon =\varepsilon_{\min}+\varepsilon_{\text{reducible}}\cdot e^{-k_{\text{apparent}}\cdot \sigma_{c}}$$

In this case, $\varepsilon_{\min}$ is the minimum out-die tablet porosity and $\varepsilon_{\text{reducible}}$ reflects the maximum possible reduction in tablet porosity. $k_{\text{apparent}}$ or the reciprocal value can be seen as an indication of the deformation resistance, but although our compression model was derived from the Heckel model, $1/k_{\text{apparent}}$ cannot be equated with the mean yield pressure $P_{Y}$.

**Fig. 2:**
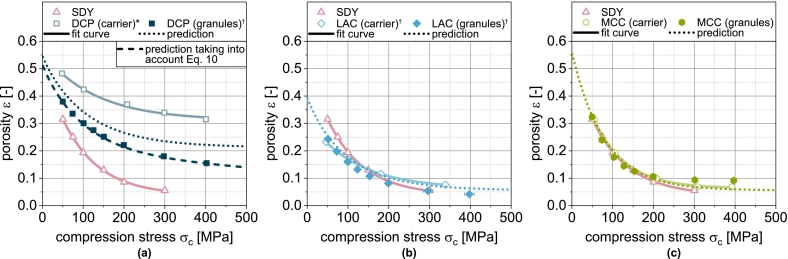
Compressibility of carrier materials DCP, LAC and MCC as well as of spray dried yeast cells (SDY) and corresponding granules. The data were fitted using Eq. 6 and predicted values were calculated according to Eq. 9. Each data point represents mean and standard deviation (n=10). ^⁎^Lubrication with 1.0 wt.-% of MgSt. ^†^Lubrication with 0.5 wt.-% of MgSt.

The application of the compression model (Eq. 6) provides an excellent fit of the experimental tablet porosities of the carrier materials DCPu^1^, LACu and MCCu (Fig. 2). The different curves for DCPu, LACu and MCCu result from the different deformation properties. Due to the brittle deformation, which is characterized by a high deformation resistance and by the fragmentation of the particles, tablets made of DCPu exhibit the lowest compressibility, i.e., the highest porosity at a given compression stress. In the case of MCCu and LACu, the compressibility is higher because the deformation resistance is lower.

The experimental compressibility data of DCPg^2^, LACg and MCCg granules predominantly show curves between those of the corresponding individual components (i.e., carrier and spray dried yeast (SDY), Fig. 2). Such an interplay was also observed during compaction of physical mixtures of different powders where a linear correlation of porosity and mass fraction of one of the components occurred (Busignies et al., 2006; Puckhaber et al., 2023). However, due to the different solid densities of the components, their volume fractions may differ significantly (see Table 1), so that the linear correlation reported when considering mass ratios (Busignies et al., 2006; Puckhaber et al., 2023) appears to be a random coincidence. Accordingly, the compressibility of physical binary powder mixtures is modeled on the basis of the volume fractions in other publications (Mazel et al., 2011; Puckhaber et al., 2023). The previously obtained fit parameters were used in analogy to these studies to predict the compressibility of DCPg, LACg and MCCg as well as the SDY. To model the compressibility of physical mixtures with n components, the solid volume fractions $\varphi_{s,i}$ of the respective components i are required:

(7) $$\varphi_{s,i}=\frac{V_{i}}{V_{\text{total}}}=\frac{\frac{x_{i}}{\rho_{s,i}}}{\sum_{i=1}^{n}\frac{x_{i}}{\rho_{s,i}}}$$

In this equation, the solid volume $V_{i}$ of the component i is set in relation to the total solid volume of the formulation $V_{\text{total}}$. $x_{i}$ is the mass fraction of component i and $\rho_{s,i}$ is the corresponding solid density. In tablets, however, the bulks of the individual components typically exhibit a certain porosity $\varepsilon_{i}$, which is not taken into account in Eq. 7. For physical mixtures, however, it is assumed that the components can be considered as bulk materials that deform independently of each other (Frenning et al., 2009) and exhibit compression stress-dependent porosity. Accordingly, the bulk volume fraction $\varphi_{i}$ of the components depends on the compression stress applied (Reynolds et al., 2017):

(8) $$\varphi_{i}=\frac{\frac{x_{i}}{\left(1-\varepsilon_{i}\right)\cdot \rho_{s,i}}}{\sum_{i=1}^{n}\frac{x_{i}}{\left(1-\varepsilon_{i}\right)\cdot \rho_{s,i}}}$$

The porosity $\varepsilon_{i}$ of the bulk of component i is obtained by a suitable compressibility model, in this case Eq. 6. For the calculation of the porosity $\varepsilon_{\text{blend}}$ of a physical mixture, a linear volumetric weighting of the porosity of the individual components is then typically carried out:

(9) $$\varepsilon_{\text{blend}}=\sum_{i=1}^{n}\varphi_{i}\cdot \varepsilon_{i}=\sum_{i=1}^{n}\varphi_{i}\cdot \left(\varepsilon_{\min,i}+\varepsilon_{\text{reducible},i}\cdot e^{-k_{\text{apparent},i}\cdot \sigma_{c}}\right)$$

**Fig. 3:**
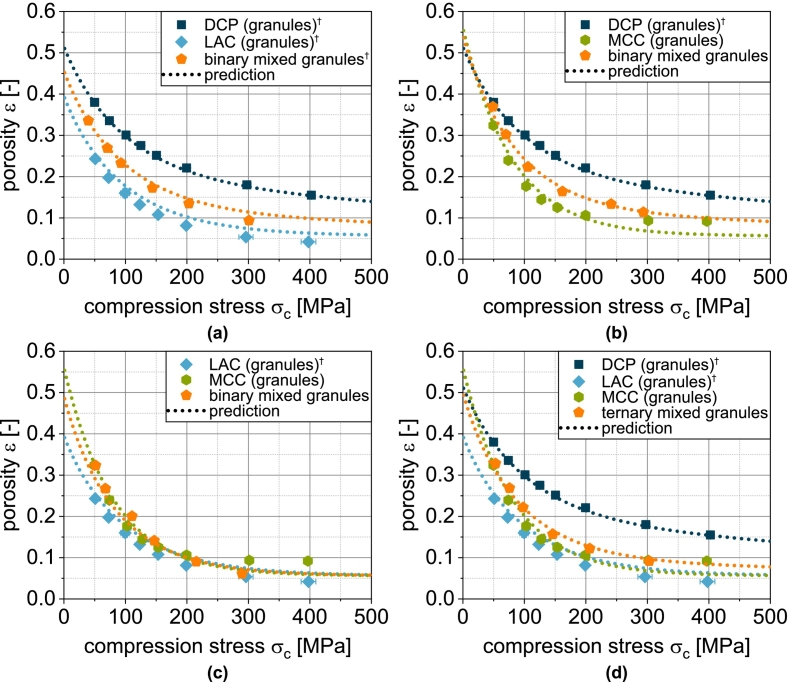
Experimental and predicted compressibility of single, binary and ternary carrier granules. Each data point represents mean and standard deviation (n=10). Predicted values were calculated according to Eq. 9 after correction of $\varepsilon_{\mathrm{DCP}}$*using Eq.*10*.*^*†*^*Lubrication with 0.5 wt.-% of MgSt.*

This model is tested for its applicability to predict the compressibility of fluidized bed granules based on the fit parameters obtained for the non-granulated carrier materials and SDY as well as their volumetric proportions in the granules. The corresponding compressibility curves of granules based on one carrier material as well as the spray-dried yeast cells are shown in Fig. 2. For LACg and MCCg, a good correlation is obtained. In the case of DCPg, however, Eq. 9 underestimates the compressibility in the entire compression stress range. Here, the assumption of two independently deforming components does not seem accurate. This can probably be attributed to the morphology of the DCP particles and the high deformation resistance of DCP, especially in comparison to SDY. Yeast and protective additives are easily deformable and are forced into the pores of the DCP during the densification of the granules, whereby the tablet porosity is reduced to lower values than the simple assumption of coincidence. In order to enable a further consideration for DCP-containing granules, the equation for calculation the compressibility was extended by a linear correction term to reduce the influence of DCP, which results from the deviation of the (extrapolated) experimental data and modeled values at $\sigma_{c}=0\;\mathrm{MPa}$ and $\sigma_{c}=400\;\mathrm{MPa}$ (Fig. 3a):

(10) $$\varepsilon_{\mathrm{DCP},\text{corrected}}=\varepsilon_{\mathrm{DCP}}\cdot \left(0.9-\frac{0.2}{400}\cdot \sigma_{c}\right)$$

**Fig. 4:**
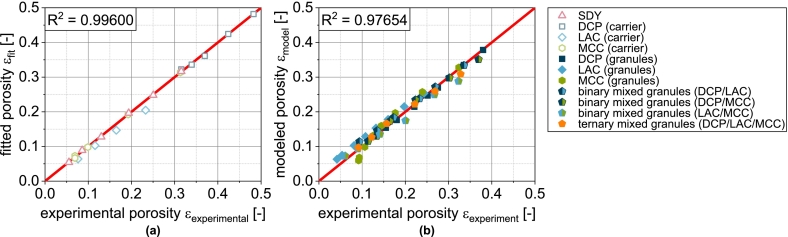
Parity plot of (a) fitted compressibility data of carrier materials and spray dried yeast cells and (b) predicted compressibility data of corresponding granules.

With this extension, the compressibility of DCPg can also be well predicted on the basis of the fit parameters of the individual components (Fig. 2a and Fig. 4b). In all subsequent considerations of granules with DCP, the corrected porosity $\varepsilon_{\mathrm{DCP},\text{corrected}}$ is always used for the DCP fraction.

In the next step, the applicability of the simple compressibility model for more complex granules based on two or three different carrier materials was evaluated by enhancing the number of components in Eq. 8. These granules exhibit compressibilities that lie between those of the corresponding single-carrier granules. Due to the higher volume fraction of LAC compared to DCP at the same mass fraction, the binary mix granules made from these carriers exhibit porosities that tend to correspond more closely to those of LACg (Fig. 3a). The same applies analogously to the combination of DCP and MCC (Fig. 3b). The volume fractions of MCC and LAC are less different due to their similar density and compressibility, which is why the granules with both excipients also show a very similar behavior (Fig. 3c). When DCP, LAC and MCC are combined, the volume fraction of DCP decreases further, which explains the higher compressibility of this ternary mix granule compared to the binary mix granules with DCP (Fig. 3d). The application of the model shows good prediction of tablet porosity for all mix granules (Fig. 3 and Fig. 4b).

Further investigations are necessary to identify the validity range of the model for granules and to correlate the deviations that were observed with DCP with measurable particle properties. In particular, studies with other brittle carrier materials such as tricalcium phosphate could be helpful in this regard. These could enable a prediction of the compressibility of binary or more complex mix granules, including those with DCP (and other brittle materials), without the need for a correction based on the single-carrier granules.

### 3.3 Modeling of compactibility

**Fig. 5:**
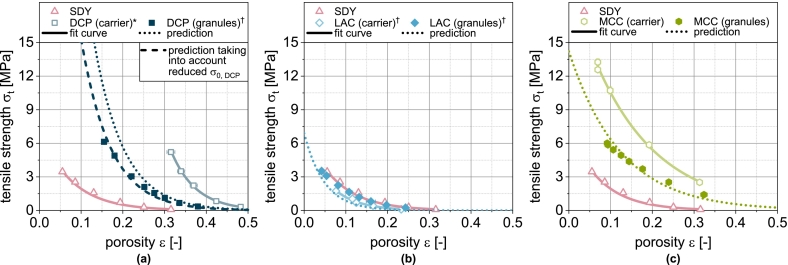
Compactibility of carrier materials DCP, LAC and MCC as well as of spray dried yeast cells (SDY) and corresponding granules. The data were fitted using Eq. 11 and predicted values were calculated according to Eq. 13. Each data point represents mean and standard deviation (n=10). ^⁎^Lubrication with 1.0 wt.-% of MgSt. ^†^Lubrication with 0.5 wt.-% of MgSt.

The porosity of tablets as a structural parameter determines their mechanical strength. The corresponding interplay of tablet porosity and tensile strength is compactibility. The non-granulated carriers DCPu, LACu and MCCu as well as SDY show different compactibilities (Fig. 5). The compactibility depends on various factors and is therefore different for different material qualities. For the grades used here, DCPu is characterized by the highest compactibility, i.e. it achieves high tensile strengths even at high porosities. Although MCCu achieves the highest tensile strengths in the compression stress range under consideration, it requires greater densification than DCPu. LACu tablets exhibit the lowest compactibility. Compactibility is typically described using the Ryshkewitch-Duckworth equation (Duckworth, 1953; Ryshkewitch, 1953):

(11) $$\sigma_{t}=\sigma_{0}\cdot e^{-k_{b}\cdot \varepsilon }$$

In this equation, $\sigma_{0}$ is the theoretical maximum tensile strength at zero porosity and $k_{b}$ is a measure of the bonding capacity. Analogous to the compressibility, the carrier materials and the spray-dried yeast cells were first fitted with this model equation. The experimental data are described by the equation with very good correlation (Fig. 5 and Fig. 7a).

In order to predict the compactibility of the granules, established models for describing physical powder mixtures are also used. The model of Etzler et al. describes the tensile strength of a mixture with a geometric mean value rule of the interactions between the particles, weighted with their surface area fraction $\varphi_{a,i}$ (Etzler et al., 2011):

(12) $$\sigma_{t,\text{blend}}=\prod_{i=1}^{n}\sigma_{t,i}^{\varphi_{a,i}}$$

The specific surface of the carrier materials DCPu, LACu and MCCu can easily be determined by gas adsorption or the external specific surface of the particles can be estimated based on the particle size distribution. After granulation, however, the surface ratios are not experimentally accessible. However, due to the formation of a layer of the granulation liquid solids on the carrier particles, the discharge of fine particles out of the granulator and the agglomeration of particles, it must be assumed that the surface area ratio changes significantly during granulation. Accordingly, the prediction of the compactibility of the granules on the basis of the external specific surface area determined by laser diffraction (data not shown) was unsuccessful. Instead, the model was applied in the following form (Etzler et al., 2011):

(13) $$\sigma_{t,\text{blend}}=\prod_{i=1}^{n}\sigma_{t,i}^{\varphi_{i}}$$

$\varphi_{i}$ is the volume fraction of component i. This special case applies when the particle sizes of the components are identical (Etzler et al., 2011). Even if this is not the case here, a very good prediction of the compactibility of the granules is obtained based on the fit parameters obtained for the non-granulated carrier materials and SDY as well as their volume fractions in the granules were, particularly for LACg and MCCg (Fig. 5).

In the case of DCPg, on the other hand, there is again a deviation between experimental and predicted data. In this case, the tablet tensile strength is overestimated by the model. For this case, the experimental data fitted for the individual components DCPu and SDY hardly overlap (very different porosity ranges). Presumably, the deviations are due to the uncertainty of $\sigma_{0,\mathrm{DCP}}$, since a considerable extrapolation is carried out here. To enable a further prediction of granules with DCP content, a correction of the model was also performed. In this case, this includes the reduction of $\sigma_{0,\mathrm{DCP}}$ for the calculation of $\sigma_{t,\text{blend}}$ from 815 to 380 MPa. The corrected value was obtained by minimizing the deviation between experimental data of DCPg and the corresponding prediction. This adjustment allows a very good description of the experimental data (Fig. 5a). In all subsequent considerations of granules with DCP, the reduced value of $\sigma_{0,\mathrm{DCP}}$ is always used.

**Fig. 6:**
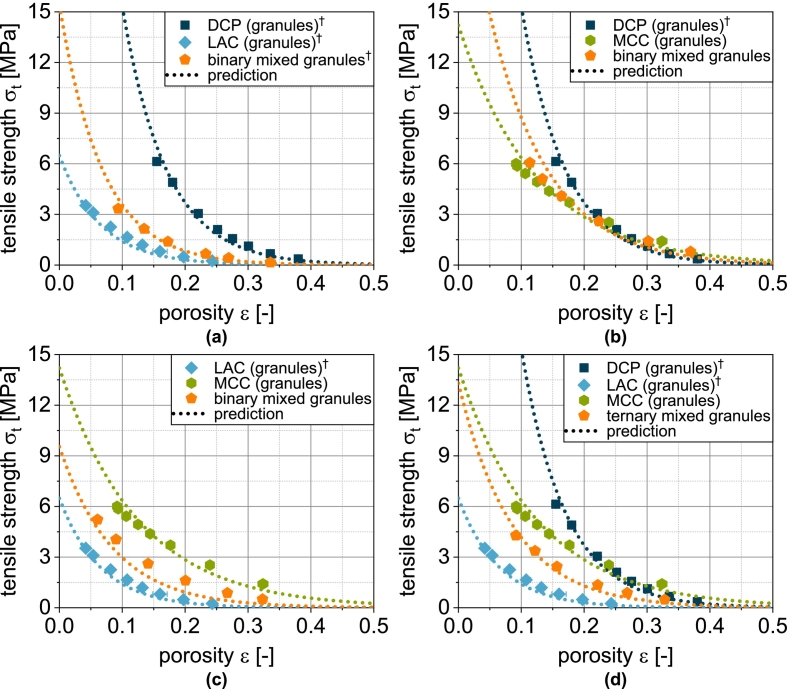
Experimental and predicted compactibility of single, binary and ternary carrier granules. Each data point represents mean and standard deviation (n=10). The prediction was calculated according to Eq. 13 with the reduced value of $\sigma_{0,\mathrm{DCP}}$*.*^*†*^Lubrication with 0.5 wt.-% of MgSt.

**Fig. 7:**
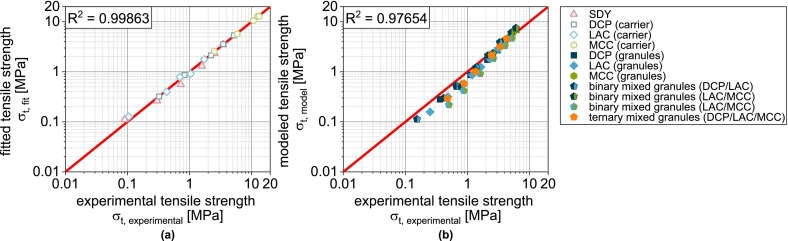
Parity plot of (a) fitted compactibility data of carrier materials and spray dried granulation liquid and (b) predicted compactibility data of corresponding granules.

In the next step, the transferability to the more complex granules based on two or three different carrier materials was tested, since the experimental data again show intermediate results. In the case of the binary mix granules DCP/LAC, it is clear that the compactibility is largely defined by the LAC content in the formulation (Fig. 6a). This can again be attributed to the low volumetric content of the DCP. The same applies to the mix granules DCP/MCC. Here, the mix granules also show compactibilities that are closer to the MCCg, whereby this applies both in the area in which the compactibility of MCCg is higher and in the area in which the compactibility of DCPg is higher (Fig. 6b). Accordingly, the mix granules also meet the intersection of the compactibility profiles of the individual granules. For the binary mixture LAC/MCC, the granules show an average compactibility between the individual granules (Fig. 6c), which can be attributed to the almost identical volume fraction of the two components. If DCP is added as an additional component (DCP/LAC/MCC) in the granulation process, the compactibility is slightly increased (Fig. 6d). However, due to the very low volume fraction of DCP, the effect is hardly noticeable. The application of the model again shows good prediction accuracy for the more complex granules (Fig. 6 and Fig. 7b). The parity diagram indicates a slight underestimation of the tensile strength at low strengths, although the logarithmic scaling of the diagram must be taken into account.

Overall, it can therefore be stated that the volume related composition of the granules has an effect on the compactibility, whereby the behavior of mix granules follows a volumetric mixing rule. Although volume-weighted mixing rules are also frequently used when modeling the compactibility of multi-component tablets (physical mixtures) (Busignies et al., 2012; Mazel et al., 2011; Puckhaber et al., 2023), this good correlation is a bit surprising as one of the basic assumptions of the model is not fulfilled. However, this only appears to have a relevant effect when DCP is used. Further investigations are necessary to identify the cause of this with certainty and to correlate it with measurable properties of the particles. These could be integrated directly into the model and make the intermediate step of correction using DCPg obsolete.

### 3.4 Modeling of tabletability

**Fig. 8:**
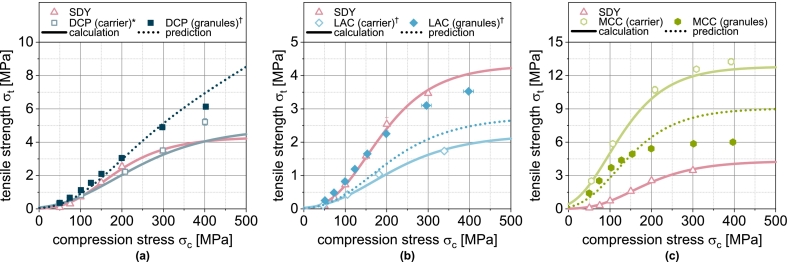
Tabletability of carrier materials DCP, LAC and MCC as well as of spray dried yeast cells (SDY) and corresponding granules. Each data point represents mean and standard deviation (n=10). Calculated data were obtained using Eq. 14 and predicted values were calculated according to Eq. 15. ^⁎^Lubrication with 1.0 wt.-% of MgSt. ^†^Lubrication with 0.5 wt.-% of MgSt.

Tabletability combines compressibility and compactibility by considering the tablet tensile strength as a function of the applied compression stress (Fig. 8). MCCu exhibits the best tabletability, but converges to a maximum tensile strength of about 14 MPa (Fig. 8c). This can be attributed to the fact that the MCCu tablets are already highly compacted at this high compression stress and further compaction is hardly possible (porosity limit value in Fig. 2). Any additional energy is introduced elastically to an increasing extent (elastic recovery in Fig. S2) and does not contribute to any further decrease in porosity and an associated increase in strength. The compressibility of the LACu tablets also flattens out slightly in the compression stress range under consideration (Fig. 2), which is why the tabletability for high compression stresses also flattens out slightly (Fig. 8b). No such behavior can be observed for the DCPu tablets, which is why the tabletability is largely linear (Fig. 8a).

Mathematically, the tabletability results from Eqs. 11 and 6 as follows:

(14) $$\sigma_{t}=\sigma_{0}\cdot e^{-k_{b}\cdot \left(\varepsilon_{\min}+\varepsilon_{\text{reducible}}\cdot e^{-k_{\text{apparent}}\cdot \sigma_{c}}\right)}$$

For the carrier materials DCPu, LACu and MCCu, as well as the spray-dried yeast cells, the tabletability results directly from the fitted compressibility and compactibility curves according to Eq. 14. The deviation between the experimental and calculated data for the non-granulated carrier materials is correspondingly small (Fig. 8). Only in the case of DCPu, the strength at a compression stress of 400 MPa is underestimated remarkable.

The experimental tabletability curves of the single carrier granules DCPg, LACg and MCCg show essentially the same trends as before. MCCg allows the highest tensile strength of about 6 MPa, but the curve flattens out significantly due to the flattening of the compressibility curve. The tablets made of LACg show a similar trend, but with lower tensile strengths compared to MCCg and with a later flattening of the curve. Nevertheless, the granulation significantly improved the overall tabletability and enabled higher tensile strengths compared to the carrier material. With DCPg, a largely linear curve can be seen at high compression stresses. A slight flattening of the curve can be attributed to the SDY content. Here, synergistic effects lead to better tabletability than with the individual components.

For multi-component systems, such as the granules in this case, Eqs. 13 and 14 lead to:

(15) $$\sigma_{t,\text{blend}}=\prod_{i=1}^{n}{\left(\sigma_{0,i}\cdot e^{-k_{b,i}\cdot \left(\varepsilon_{\min,i}+\varepsilon_{\text{reducible},i}\cdot e^{-k_{\text{apparent},i}\cdot \sigma_{c}}\right)}\right)}^{\varphi_{i}}$$

For the granules, the correlation between experiment and model is somewhat different. While the tabletability of DCPg is well predicted by Eq. 14, the strength is underestimated in the case of LACg and overestimated in the case of MCCg at compression stresses above 200 MPa. By using compressibility and compactibility data for the prediction of tabletability, initially small deviations are amplified and cause the sometimes poorly fitting curves.

**Fig. 9:**
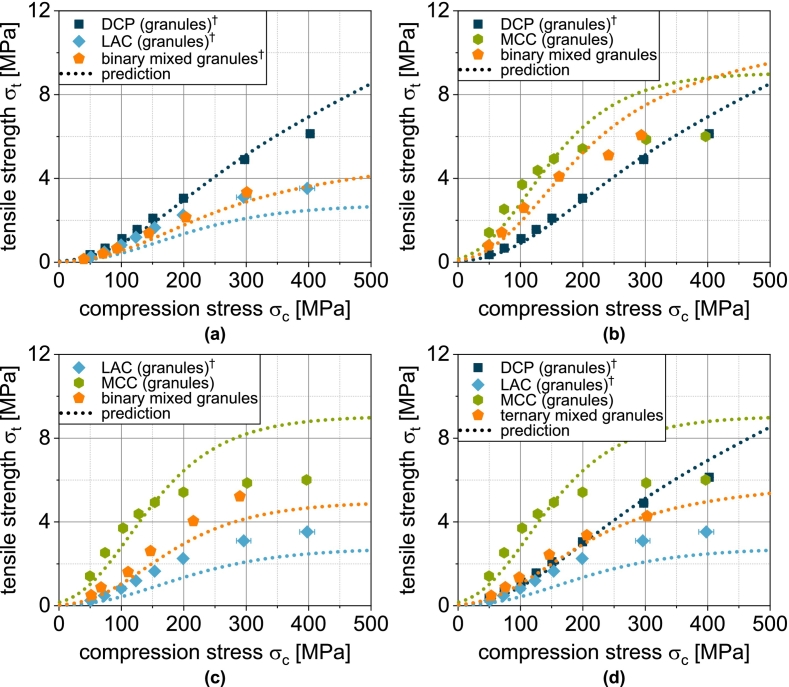
Experimental and predicted tabletability of single, binary and ternary carrier granules. Each data point represents mean and standard deviation (n=10). The prediction was calculated according to Eq. 15. ^†^Lubrication with 0.5 wt.-% of MgSt.

The tabletability of the mix granules DCP/LAC corresponds to the tabletability of LACg (Fig. 9a). The low volume fraction of DCP has hardly any effect here. In case of the mix granules DCP/MCC, DCP leads to a certain reduction in tabletability despite its low volume fraction (Fig. 9b). However, it is also clear that the tabletability profile of the mix granules flattens out less. This can be attributed to the fact that the deformation of the brittle DCP becomes increasingly important at high compression stresses. The mix granules LAC/MCC contain a larger proportion of LAC by volume than MCC in the mix granules DCP/MCC. Accordingly, the tabletability of the mix granules is greatly reduced here compared to MCCg (Fig. 9c). The largely linear course of the tabletability here is a consequence of the higher deformation resistance of LACg compared to MCCg. Substantial densification occurs here even at higher compression stresses and allows an increase in strength. If an additional component is added to the mix granules (DCP/LAC/MCC), a greater reduction in tabletability can be observed as a consequence of the lower volume fraction of MCC (Fig. 9d).

**Fig. 10:**
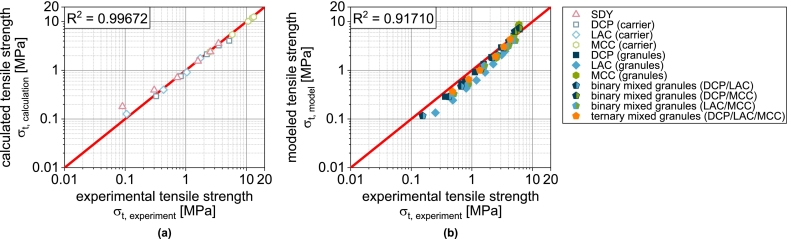
Parity plot of (a) calculated compactibility data of carrier materials and spray dried yeast cells and (b) predicted compactibility data of corresponding granules.

If the model is also applied to more complex granules based on two or three carriers, regardless of the sometimes poor fit between the experimental and predicted tensile strengths of the single carrier granules (Fig. 8), the curves of the experimental data can still be predicted well in some cases (Fig. 8 and Fig. 10b). Overall, however, an underestimation of the tensile strength can be observed (Fig. 10b). Specific interactions therefore improve the tabletability of the granules compared to the simple model of several independently behaving powders. However, in view of the fact that the powders are obviously not present individually, the agreement between the experimental and predicted tabletability curves is remarkable. The above-mentioned necessary further investigations to clarify the deviating behavior of DCP (compressibility and compactibility) should reveal parameters that are also determined by all other components and should be included in the compressibility and compactibility modeling. An even more precise prediction of these properties would also improve the prediction accuracy of tabletability.

### 3.5 Microbiological tablet properties

In addition to the physical-mechanical tablet properties, the microbiological properties are of crucial importance for probiotic tablets. First, the survival of the yeast cells during tableting is considered in order to assess the influence of the granule formulation on the damage to the microorganisms during this process step. Subsequently, the absolute viability is evaluated as a measure of the number of viable yeast cells contained in a tablet. This enables the inclusion of different survival rates during granulation depending on the formulation used for the overall comparison of the suitability of the different formulations.

**Fig. 11:**
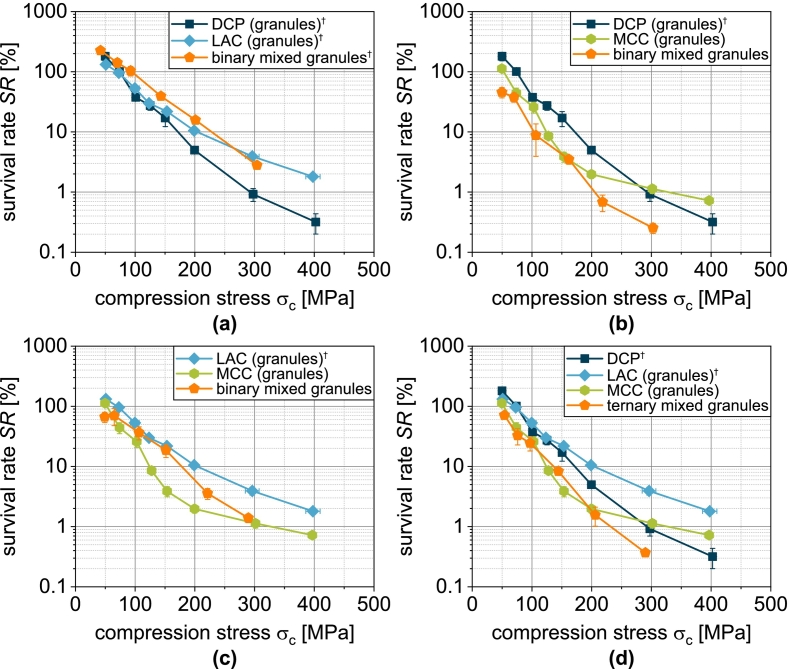
Survival of yeast cells related to the applied compression stress during tableting of granules based on one single carrier material as well as granules based on mixtures of the carrier materials (binary and ternary). Data points show mean and standard deviation (n=3). ^†^Lubrication with 0.5 wt.-% of MgSt.

Almost all formulations show survival rates above 100 % at low compression stresses (Fig. 11). This means that a higher number of living microorganisms is found when resuspending the tablets than when resuspending the corresponding mass of granules. This is an effect that is almost always observed by the same authors and has also been described by other authors. In a previous publication, this phenomenon was discussed in detail and finally attributed to the different reconstitution kinetics experienced by the microorganisms during rapid moistening of the granules compared to the tablet (Vorländer et al., 2023b). At higher compression stresses, however, the mechanical stresses clearly outweigh this effect and cause the sharp decrease in the survival rate (Vorländer et al., 2023b), which is also evident here for all formulations (Fig. 11).

In contrast to the physical-mechanical tablet properties, the survival of the microorganisms as a function of the applied compression stress for the mix granules does not necessarily show intermediate curves (Fig. 11). While slightly higher survival rates can be achieved with the combination DCP/LAC LAC (Fig. 11a), the combination of DCP/MCC is detrimental to survival (Fig. 11b). When comparing the curves of the individual granules (Fig. 11d), it becomes clear that the use of MCCg is associated with the lowest survival. This was associated with the easy plastic deformation of MCC in earlier studies by the same authors, whereby compressive and shear stresses are transferred particularly effectively to the microorganisms and lead to their mechanical damage and inactivation. The same applies to the mix granules with MCC, whereby the addition of the more brittle deforming materials seems to lead to an intensification of the damage, especially at high compression stresses. It is conceivable that microorganisms at the interface between the ductile deforming MCC and the stiff DCP are exposed to particularly high stresses, since on the one side MCC exerts shear forces on the cells, but on the other side is a rigid material. Accordingly, its deformation is negligible and the yeast cells are more strongly deformed. In addition, the DCP particles are characterized by a fissured surface, which can lead to local stress peaks and cutting, which further impairs survival. In order to understand more precisely how the yeast cells are inactivated, it is necessary to consider the porosity.

**Fig. 12:**
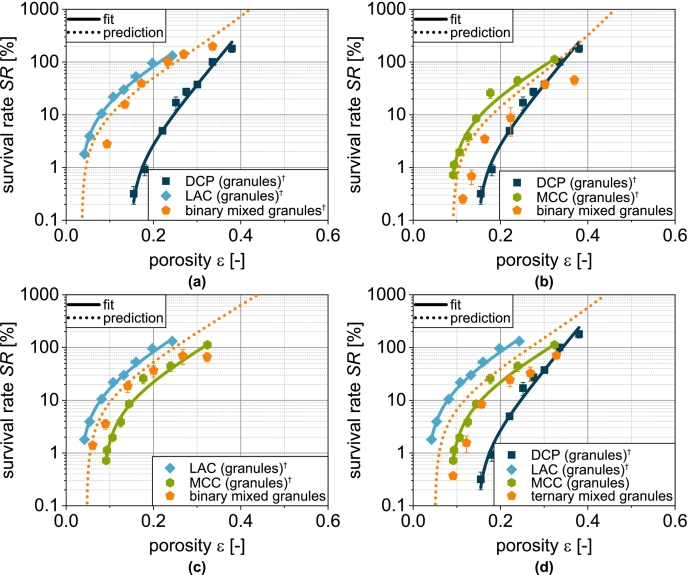
Survival of yeast cells related to tablet porosity for tablets based on granules based on one single carrier material as well as granules based on mixtures of the carrier materials (binary and ternary). Data points show mean and standard deviation (n=3). The data were fitted using Eq. 16 and predicted values were calculated according to Eq. 17. ^†^Lubrication with 0.5 wt.-% of MgSt.

Regardless of the formulation, a reduction in porosity is accompanied by a reduction in survival (Fig. 12). This is obvious, as a lower porosity can be assumed to increase the effect of compression and shear on the microorganisms. Depending on the formulation, however, different survival rates are observed for the same porosity. These are highest for tablets made of LACg and lowest for tablets made of DCPg. Tablets made of MCCg and the mix granules show intermediate survival rates. For the combination LAC/DCP, DCP hardly influences the porosity-related survival (Fig. 12a). This can be attributed to the low volumetric proportion of DCP in the formulation.

**Fig. 13:**
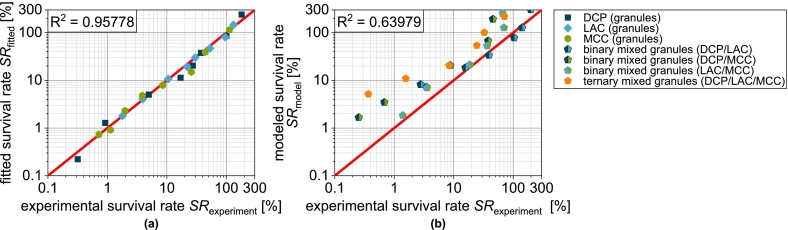
Parity plot of (a) fitted survival rate of single carrier granules and (b) predicted survival rate of granules based on two and three carrier materials.

To illustrate this, the experimental data of single carrier granules were fitted with the following function (Vorländer et al., 2025b):

(16) $$\mathrm{SR}\left(\varepsilon \right)=a+b\cdot e^{-k_{\mathrm{SR},\varepsilon }\cdot \varepsilon }$$

with a and b as empirical fit parameter representing the offset and initial value of the curve and $k_{\mathrm{SR},\varepsilon }$ as tablet porosity-related inactivation rate. This equation provides a good description of the experimental data (Fig. 12 and Fig. 13a). Thus, the survival rate for granules based on several carrier materials can be determined using the following equation:

(17) $$SR_{\mathrm{mix}}\left(\varepsilon \right)=\sum_{i=1}^{n}\varphi_{i}\cdot {\mathrm{SR}}_{i}\left(\varepsilon \right)$$

The assumption here is a combination of the different granules based on a single carrier material. In this case, $\varphi_{i}$ describes the volume fraction of the combined granules based on a single carrier material (two for the binary mix granules and three for the ternary mix granules) assuming an even distribution of the solids of the granulation liquid on the different carrier materials. ${\mathrm{SR}}_{i}\left(\varepsilon \right)$ is the porosity-dependent survival rate of the respective single carrier granules and is represented by Eq. 16. The survival rates calculated in this way show intermediate curves for the binary and ternary carrier granules (Fig. 12). In the case of the binary mixture LAC/DCP, a comparatively good prediction of the survival rate is possible (Fig. 12a). Also for LAC/MCC, the survival rate can be predicted well in a certain porosity range (Fig. 12c). Low compression stresses with high porosity are thereby characterized by a larger deviation. With the combination DCP/MCC (Fig. 12b) or all substrate materials (DCP/LAC/MCC, Fig. 12d), the experimental survival rates can only be predicted inadequately by the model and are always overestimated (Fig. 13b). This can possibly be attributed to the fact that the porosity distribution in the tablet is not homogeneous and locally higher porosities occur in the area of DCP particles and, conversely, other areas and the cells present there are more densely compressed.

**Fig. 14:**
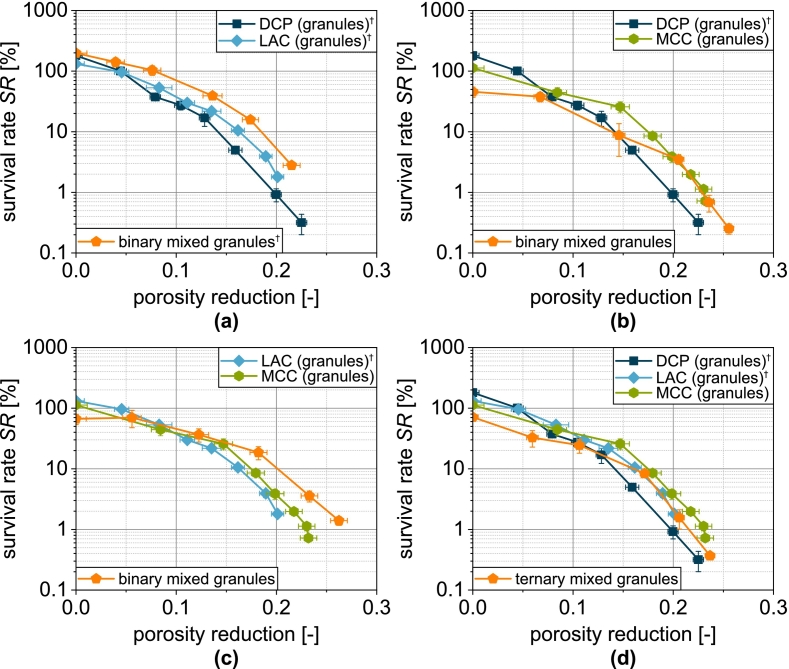
Survival of yeast cells as a function of porosity reduction during compression of granules based on one single carrier material as well as granules based on mixtures of the carrier materials (binary and ternary). Data points show mean and standard deviation (n=3). ^†^Lubrication with 0.5 wt.-% of MgSt.

In a previous publication by the same authors, an attempt was made to calculate, under simplifying assumptions, which porosity should be present in the DCP fraction and which in the fraction of yeast cells and protectants (Vorländer et al., 2023b). However, as too little information is available, the result is not very meaningful. In another approach, however, it was found that the porosity change during compaction correlates with survival across materials, as it appears to be a better measure of the shear and compressive stress on the microorganisms (Vorländer et al., 2025a; Vorländer et al., 2023d; Vorländer et al., 2023b). This was also investigated here for the mix granules and, in all cases, a good agreement was found (Fig. 14). This confirms the previously found correlation also for more complex formulations. For the best possible survival, the porosity change should be as small as possible and in particular not exceed 0.15, as the decrease becomes even greater above this value. Nevertheless, tablets must be produced with sufficient mechanical strength. Formulations that enable high tablet tensile strengths even with a low porosity reduction are advantageous here. The strength range from 1.0 to 1.7 MPa is of particular interest (Pitt and Heasley, 2013; Vorländer et al., 2020).

To assess this, survival can be considered as a function of the tensile strength of the tablets achieved (Vorländer et al., 2025b):

(18) $$\mathrm{SR}\left(\sigma_{t}\right)=SR_{\sigma_{t}=0}\cdot e^{-k_{\mathrm{SR},\sigma_{t}}\cdot \sigma_{t}}$$

$SR_{\sigma_{t}=0}$ is the theoretical survival rate at zero tablet tensile strength and $k_{\mathrm{SR},\sigma }$ the tablet tensile strength-related inactivation rate. The experimental data of the single carrier granules can be described well overall by this equation, even if the $R^{2}=0.87018$ is significantly lower than for the previous fits (Fig. 16a). This is mainly due to the fact that the experimental data do not form ideal straight lines in the logarithmic plot. Although a fit function with additional parameters could enable a better correlation, the meaning of the additional parameters would be unclear. For this reason, the proposed model is used for further analysis. Analogous to the previous procedure, the survival rate for the mix granules is calculated accordingly by volumetric weighting of the single carrier granules:

(19) $$SR_{\mathrm{mix}}\left(\sigma_{t}\right)=\sum_{i=1}^{n}\varphi_{i}\cdot {\mathrm{SR}}_{i}\left(\sigma_{t}\right)$$

$\varphi_{i}$ again describes the volume fraction of the combined single carrier granules assuming a uniform distribution of the solids from the granulation liquid on the different carrier materials. ${\mathrm{SR}}_{i}\left(\sigma_{t}\right)$ is the tensile strength-related survival rate of the respective single carrier granules and can be calculated by Eq. 18.

**Fig. 15:**
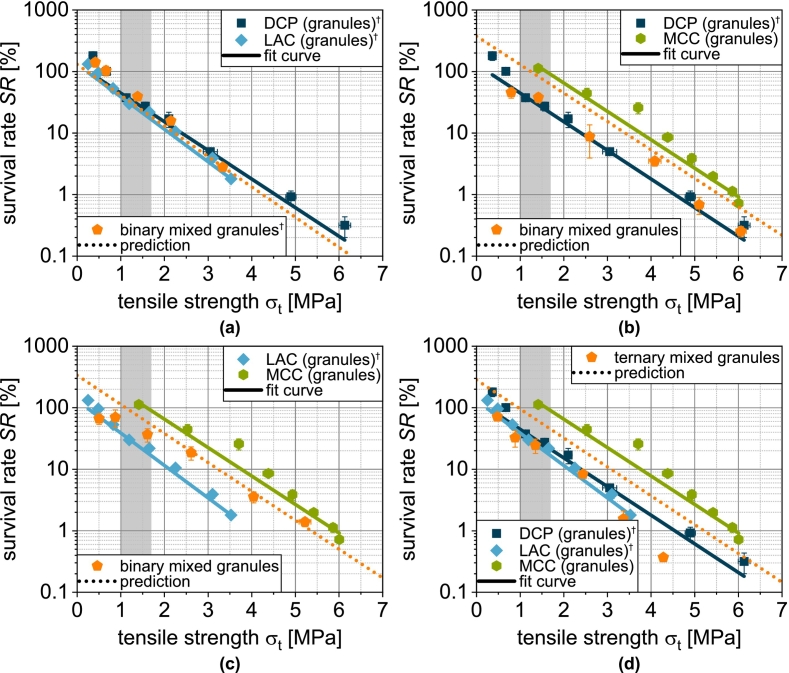
Survival of yeast cells as a function of tensile strength of tablets made of granules based on one single carrier material as well as granules based on mixtures of the carrier materials (binary and ternary). Data points show mean and standard deviation (n=3). The data were fitted using Eq. 18 and predicted values were calculated according to Eq. 19. The target tensile strength is highlighted in gray. ^†^Lubrication with 0.5 wt.-% of MgSt.

DCPg and LACg show almost identical curves, as does the binary mixture DCP/LAC (Fig. 15a). In the case of DCP/MCC, the experimental survival rate is almost identical to that of DCPg. Despite the low volume fraction, the DCP has a determining and negative impact in this case (Fig. 15b). The model applied cannot represent this effect. If, on the other hand, MCC is combined with LAC, an intermediate behavior becomes visible experimentally and in the model (Fig. 15c). However, this means that the combination remains disadvantageous compared to MCCg. If DCP is also used in a ternary mixture, this again has a significant negative effect on survival and the modeled and experimental survival rate drift apart (Fig. 15d).

**Fig. 16:**
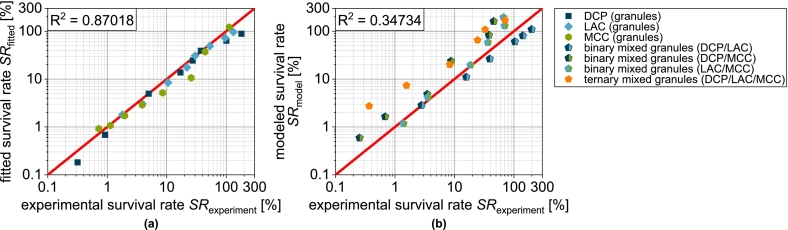
Parity plot of (a) fitted survival rate of single carrier granules and (b) predicted survival rate of granules based on two and three carrier materials.

The correlation between experimental and modeled data is correspondingly low (Fig. 16b). Further investigations are therefore definitely required to identify and quantify additional damage mechanisms, which will then enable a reliable prediction of the survival rate in more complex models.

**Fig. 17:**
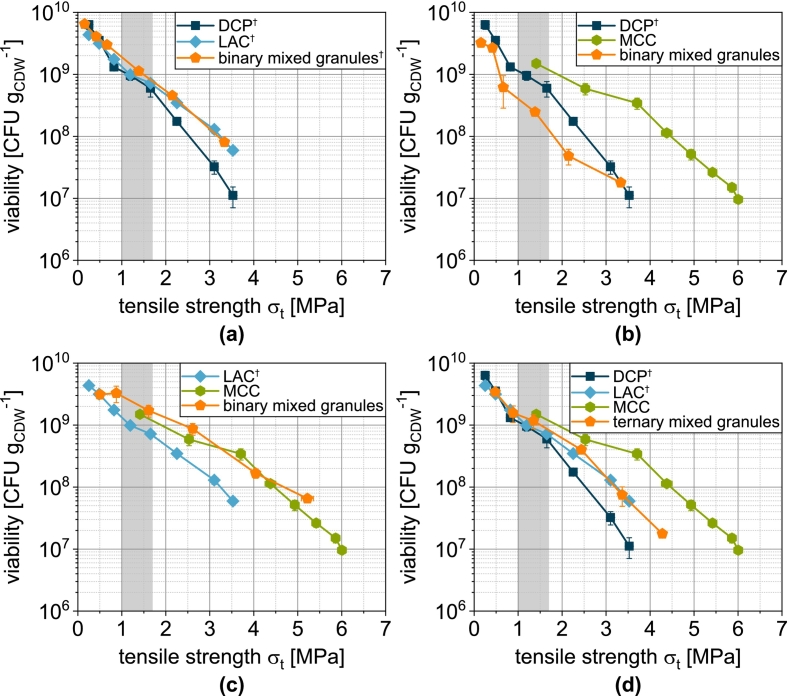
Viability of yeast cells as a function of tensile strength of tablets made of granules based on one single carrier material as well as granules based on mixtures of the carrier materials (binary and ternary). Data points show mean and standard deviation (n=3). The target tensile strength is highlighted in gray. ^†^Lubrication with 0.5 wt.-% of MgSt.

**Fig. 18:**
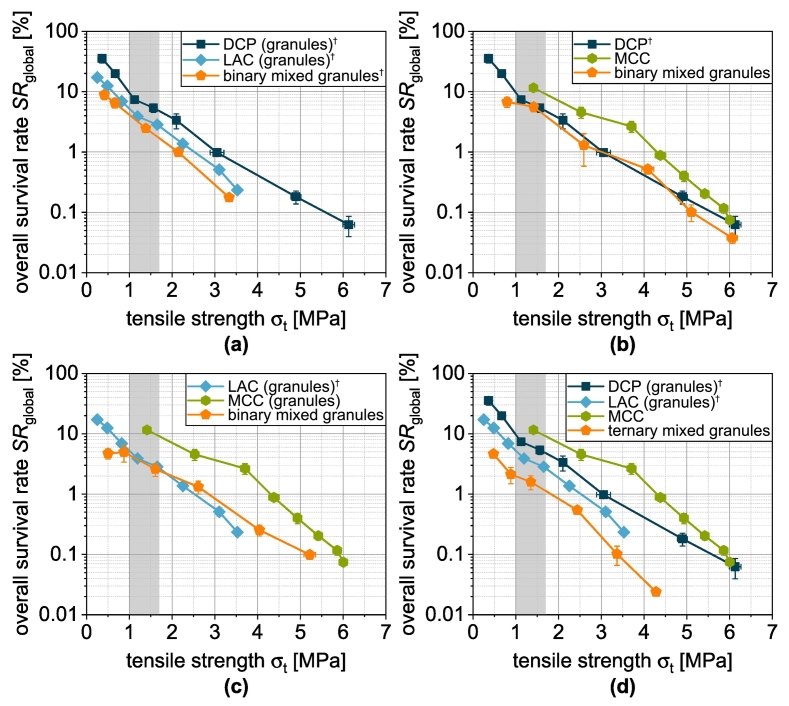
Overall survival rate of yeast cells as a function of tensile strength of tablets made of granules based on one single carrier material as well as granules based on mixtures of the carrier materials (binary and ternary). Data points show mean and standard deviation (n=3). The target tensile strength is highlighted in gray. ^†^Lubrication with 0.5 wt.-% of MgSt.

Since not only the survival during tableting but also the achievable dose is decisive for the final evaluation of the tablets, the absolute viabilities must also be considered (Fig. 17). These viabilities differ from the survival during tableting because different survival rates occurred during granulation depending on the formulation as shown in section 3.1, which can be attributed to different volume flows and slightly different bed temperatures (Vorländer et al., 2023a). The binary mixture LAC/MCC seems to be advantageous, which is due to a good interplay of survival during granulation and compression as well as the tabletability of these granules (Fig. 17c). However, it must be taken into account that different initial counts of colony forming units of the biomass used for granulation limit the comparability of the curves. To enable a cross-process comparison, the overall survival rate $SR_{\text{overall}}$ must therefore also be calculated and analyzed (Fig. 18):

(20) $$SR_{\text{overall}}=SR_{\text{granulation}}\cdot SR_{\text{tableting}}$$

Here, MCCg proved to be the clear favorite. The high tensile strength-specific survival rates during tableting more than compensate for the low survival rate during granulation (lowest survival rate of the single carrier granules). The mix granules always show a poorer performance than one or more of the corresponding single carrier granules, which can be attributed to the low survival rate during granulation.

## 4 Conclusions

In the present study, it was examined to what extent the combination of carrier materials with different deformation characteristics influences the survival of yeast cells during tableting and the physical-mechanical tablet properties. The physical-mechanical tablet properties of the mix granules showed intermediate values compared to the corresponding single carrier granules without pronounced synergies of the combination. This suggested the application of common mixing rules for the prediction of tablet properties such as porosity and tensile strength based on tablet data of the individual carrier materials and the spray-dried yeast cells. This showed that the physical-mechanical tablet properties of the tableted single, binary and ternary granules can largely be predicted by applying common volume-weighted mixing rules. Only in the case of DCP, the granulation process leads to structural changes that require an extension of the simple model equations. Further investigations should identify measurable particle properties that extent the model to be applicable generally. Possibly, taking into account the hardness and the *E*-modulus of the different materials could improve the applicability of the model. Volume-weighted mixing rules were also used to predict the survival of the microorganisms during tableting of the binary and ternary mix granules. For technical reasons, the survival rates obtained during tableting of the single carrier granules were used as a starting point. Despite the clearly more pronounced similarity of these granules instead of the non-granulated carriers to the mix granules, the prediction accuracy is low.

Overall, the prediction of the physical-mechanical tablet properties based on the carrier tableting properties should make the production and tableting of the single carrier granules as well as the associated tablet characterization obsolete in the future. Further investigations could and should aim at predicting survival based on predicted tablet porosities and associated porosity reduction during densification.

## CRediT authorship contribution statement

**Karl Vorländer:** Writing – original draft, Visualization, Validation, Methodology, Investigation, Funding acquisition, Formal analysis, Data curation, Conceptualization. **Lukas Bahlmann:** Writing – review & editing, Validation, Methodology, Investigation, Formal analysis, Data curation. **Arno Kwade:** Writing – review & editing, Supervision, Resources, Project administration, Funding acquisition, Conceptualization. **Jan Henrik Finke:** Writing – review & editing, Supervision, Resources, Project administration, Funding acquisition, Conceptualization. **Ingo Kampen:** Writing – review & editing, Supervision, Resources, Project administration, Funding acquisition, Conceptualization.

## Declaration of competing interest

The authors declare the following financial interests/personal relationships which may be considered as potential competing interests:

Karl Vorlaender reports financial support was provided by German Research Foundation. Karl Vorlaender reports article publishing charges was provided by Project DEAL. Karl Vorlaender reports equipment, drugs, or supplies was provided by Chemische Fabrik Budenheim KG. Karl Vorlaender reports equipment, drugs, or supplies was provided by J Rettenmaier and Sons. Karl Vorlaender reports equipment, drugs, or supplies was provided by MEGGLE GmbH & Co. KG. If there are other authors, they declare that they have no known competing financial interests or personal relationships that could have appeared to influence the work reported in this paper.

## Data Availability

Data will be made available on request.

## Appendix A Supplementary data

Supplementary material Image 1

## Footnotes

1 The suffix “u” is used to label the ungranulated carrier material.

2 The suffix “g” is used to label the granules.
