# Supplementary material for: Modeling of physical-mechanical and microbiological properties of tablets made of complex fluidized bed granules containing living yeast cells using common mixing rules
Source: Int J Pharm X. 2025 Oct 23;10:100423. doi: 10.1016/j.ijpx.2025.100423 (PMC12596667; doi:10.1016/j.ijpx.2025.100423)
Supplement: Supplementary file 1 — Supplementary material [file mmc1.pdf]

Supplementary Material

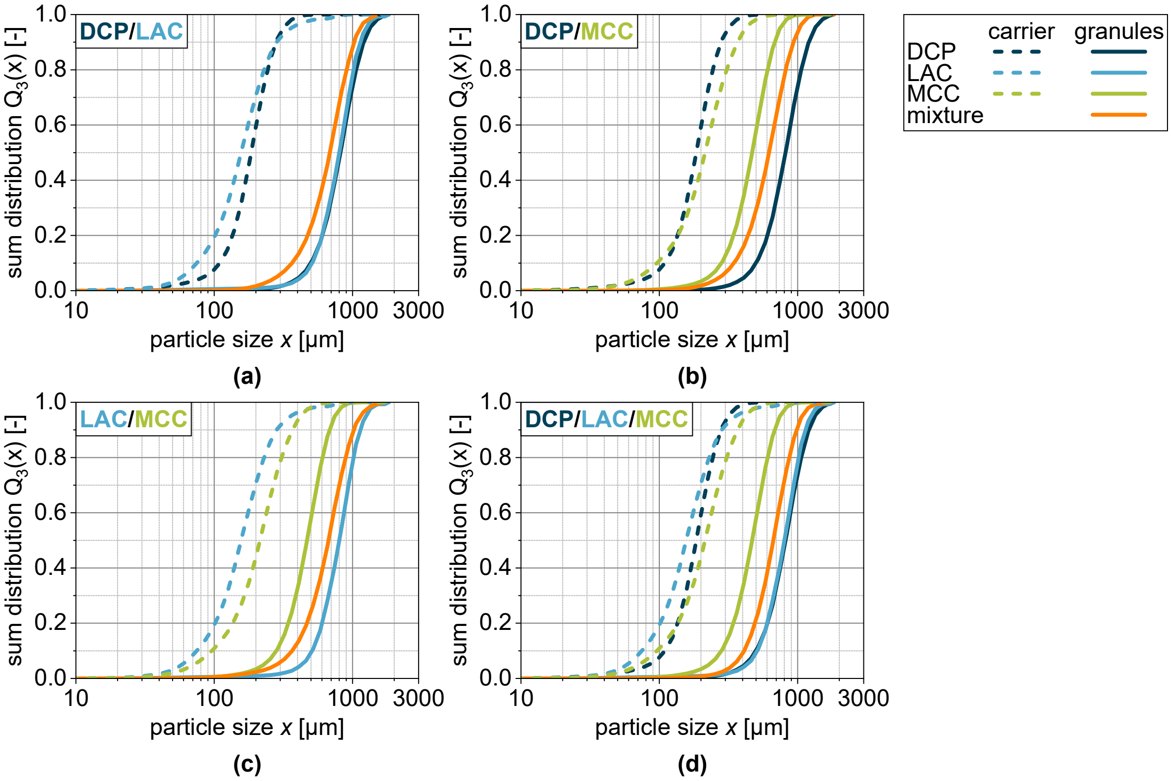

Figure S1: Particle size distributions of carrier particles DCP, LAC and MCC as well as corresponding binary mixtures (a) – (c) and ternary mixture (d).

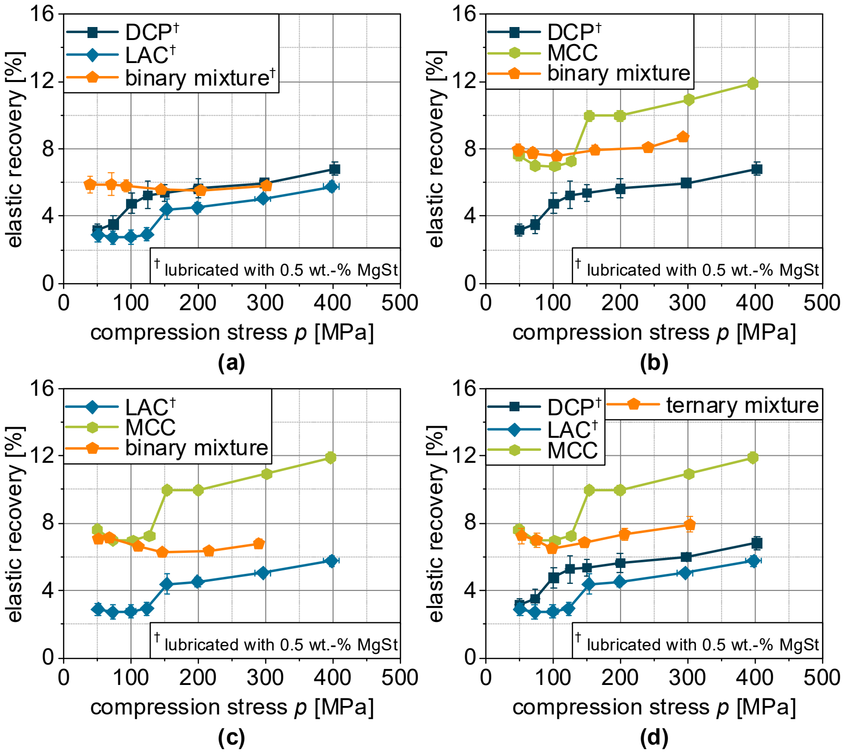

Figure S2: Elastic recovery of granules based on one single carrier material as well as granules based on mixtures of the carrier materials (binary and ternary). Data points show mean and standard deviation ($n = 10$). †Lubrication with 0.5 wt.-% of MgSt.
